# VA-TIRFM-based SM kymograph analysis for dwell time and colocalization of plasma membrane protein in plant cells

**DOI:** 10.1186/s13007-023-01047-5

**Published:** 2023-07-08

**Authors:** Bodan Su, Anqi Wang, Daoxin Xie, Xiaoyi Shan

**Affiliations:** MOE Key Laboratory of Bioinformatics, Tsinghua-Peking Joint Center for Life Sciences, and School of Life Sciences, Tsinghua University, Beijing, 100084 China

## Abstract

**Background:**

The plasma membrane (PM) proteins function in a highly dynamic state, including protein trafficking and protein homeostasis, to regulate various biological processes. The dwell time and colocalization of PM proteins are considered to be two important dynamic features determining endocytosis and protein interactions, respectively. Dwell-time and colocalization detected using traditional fluorescence microscope techniques are often misestimated due to bulk measurement. In particular, analyzing these two features of PM proteins at the single-molecule level with spatiotemporal continuity in plant cells remains greatly challenging.

**Results:**

We developed a single molecular (SM) kymograph method, which is based on variable angle-total internal reflection fluorescence microscopy (VA-TIRFM) observation and single-particle (co-)tracking (SPT) analysis, to accurately analyze the dwell time and colocalization of PM proteins in a spatial and temporal manner. Furthermore, we selected two PM proteins with distinct dynamic behaviors, including AtRGS1 (Arabidopsis regulator of G protein signaling 1) and AtREM1.3 (Arabidopsis remorin 1.3), to analyze their dwell time and colocalization upon jasmonate (JA) treatment by SM kymography. First, we established new 3D (2D+t) images to view all trajectories of the interest protein by rotating these images, and then we chose the appropriate point without changing the trajectory for further analysis. Upon JA treatment, the path lines of AtRGS1-YFP appeared curved and short, while the horizontal lines of mCherry-AtREM1.3 demonstrated limited changes, indicating that JA might initiate the endocytosis of AtRGS1. Analysis of transgenic seedlings coexpressing AtRGS1-YFP/mCherry-AtREM1.3 revealed that JA induces a change in the trajectory of AtRGS1-YFP, which then merges into the kymography line of mCherry-AtREM1.3, implying that JA increases the colocalization degree between AtRGS1 and AtREM1.3 on the PM. These results illustrate that different types of PM proteins exhibit specific dynamic features in line with their corresponding functions.

**Conclusions:**

The SM-kymograph method provides new insight into quantitively analyzing the dwell time and correlation degree of PM proteins at the single-molecule level in living plant cells.

**Supplementary Information:**

The online version contains supplementary material available at 10.1186/s13007-023-01047-5.

## Background

Proteins always remain in two fundamentally different environments of cells, either embedded in membranes or an aqueous phase. The dynamic properties of proteins within cells play a crucial role in determining their biological function [1]. To elucidate the cytological features of protein dynamic behaviors, several fluorescence microscopy imaging techniques have been developed [2]. However, a hyperfine structure or single-molecule particle might be imaged as a blurry spot due to the inappropriate optical resolution of the microscope. In addition, the lengthy exposure time of neighboring frames in a typical video sequence recorded by traditional fluorescence microscopy leads to limited available temporal information for quantitative image analysis.

In recent years, advanced fluorescence microscopy has been widely used to trace the spatiotemporal behaviors of particles and output time-lapse images at millisecond intervals [3, 4]. The original fluorescence timeseries data can be transformed into a specialized type of spatiotemporal representation by kymography, an image processing method, to accurately analyze the dynamic features of hyperfine structures such as the microtubule, actin, axon, and receptor protein [5–8]. Kymography generates a two-dimensional analog by plotting time along a spatial axis (2D+t) to describe the vertical motion of interest particles [9–11]. Hereafter, this method developed rapidly to analyze mitosis, meiosis, and filament protein traffic in mammal and yeast cells [12–15]. However, the application of kymography in plant cells remains challenging.

With the development of fluorescence microscopy, variable angle-total internal reflection fluorescence microscopy (VA-TIRFM) has made it possible to investigate the dynamic characteristics of plasma membrane (PM) proteins at the nanosecond and nanometer scales in living plant cells [1, 16–20]. Based on VA-TIRFM, researchers have revealed that the dynamics and assembly of PM proteins, such as oligomerization and stoichiometry, function as key determinants of many biological processes [1, 16–20]. However, the dwell time and colocalization of PM proteins are two important but easily misinterpreted dynamic parameters. Dwell time of active PM proteins indicates the initial for endocytosis events and the colocalization degree could envision the possibility of protein–protein interactions [2].

Here, we propose the combination of two approaches, VA-TIRFM and kymography, to establish a single-molecule (SM) kymograph method to analyze PM protein dynamics in living cells, with special emphasis on dwell time and colocalization. Although we focus on the dynamic features of plant PM proteins, this method can in principle be used to record the mobility of highly fine structures in any cell types and to assess the relevance of various cellular organelles in signal transduction.

## Results

### TIRFM-tracked protein dynamics

In this study, we selected two PM proteins with distinct dynamic behaviors, namely AtRGS1 (Arabidopsis regulator of G protein signaling 1) and AtREM1.3 (Arabidopsis remorin 1.3), to trace the movements of single particles on the PM by VA-TIRFM [19, 21, 22]. AtRGS1, a protein with seven N-terminal transmembrane helices, could traffic from the PM to the endosome upon different treatments such as d-glucose, flg22, and jasmonate (JA) [21, 22]. However, AtREM1.3, a protein resident in lipid rafts of PM, presents lateral and temporal stability in the PM [19].

Transgenic *Arabidopsis* seedlings expressing C-terminal YFP-tagged AtRGS1 and N-terminal mCherry-labeled AtREM1.3 driven by their native promoters in the corresponding mutant background were used in the VA-TIRFM analysis. The expression level of the interest genes in the transgenic seedlings should be in line with that of the wild-type seedlings. Recent studies have shown that most plant PM proteins transfer into clathrin-coated pits, which have a residence time on the PM of about 30 s. Therefore, time-lapse series images of PM proteins were set at up to 200 images and a 150-ms exposure time (up to 30 s per recording) per sequence to ensure that all the dynamic features of the interested proteins could be recorded. The raw image data were stored in TIFF format for subsequent kymograph analysis.

**Fig. 1:**
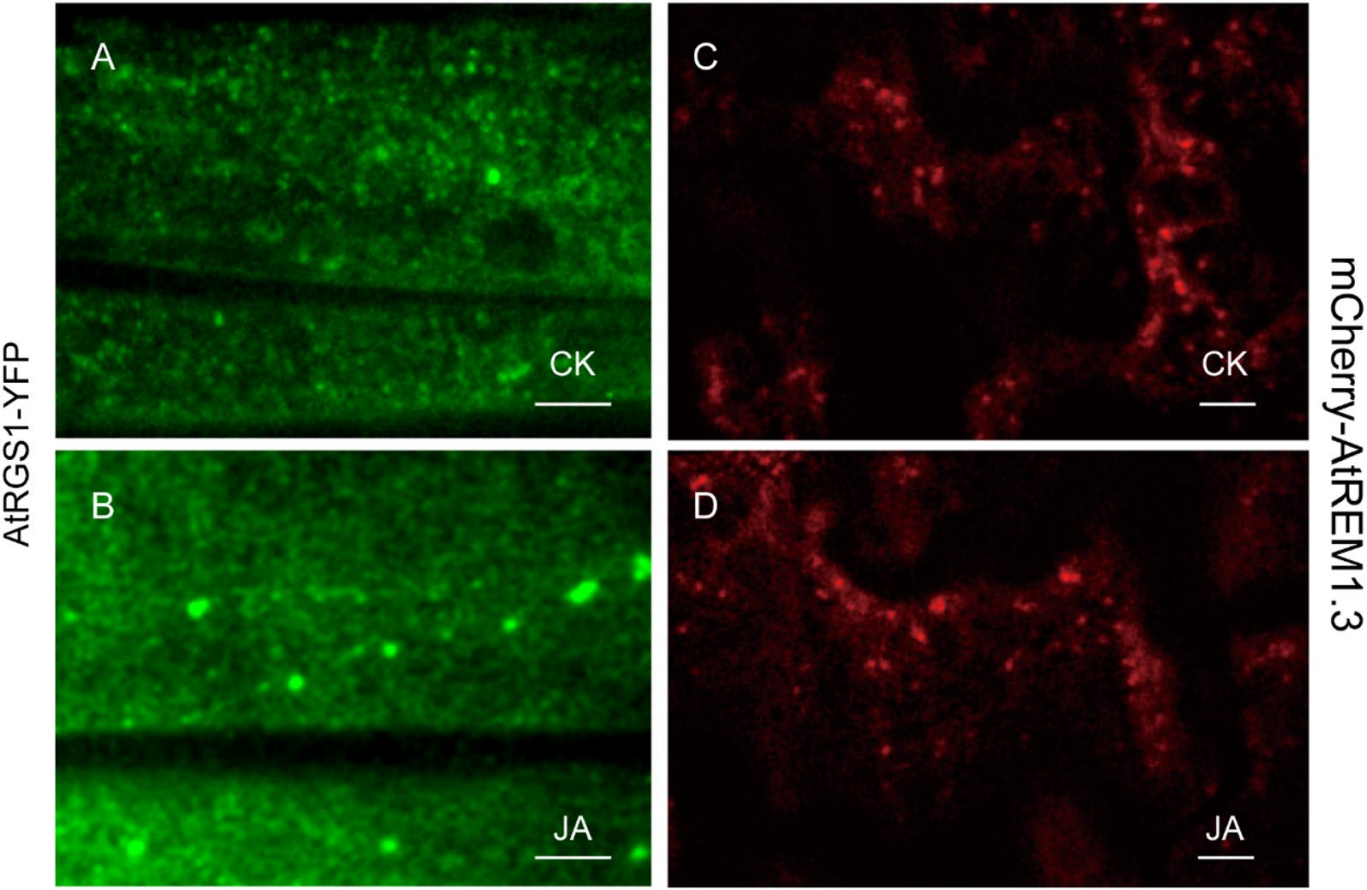
Distribution of the AtRGS1-YFP and mCherry-AtREM1.3 protein on the PM under different conditions. **A**, **B** Representative single-particle images of AtRGS1-YFP in *Arabidopsis* hypocotyl cells by VA-TIRFM. The 6-day-old transgenic seedlings expressing AtRGS1-YFP were treated with ½ MS liquid medium (CK) and 100 μM MeJA (JA) for 8 h; **C**, **D** representative single-particle images of mCherry-AtREM1.3 in *Arabidopsis* leaf epidermal cells by VA-TIRFM. The 6-day-old seedlings expressing mCherry-AtREM1.3 were treated with ½ MS liquid medium (CK) or with 100 μM MeJA (JA) for 8 h. Bar = 2 μm in **A**–**D**

In addition to treatment with a half-strength Murashige & Skoog (MS) liquid medium as a control, we further used JA as an activating agent to induce the endocytosis of AtRGS1. The typical images of AtRGS1-YFP and mCherry-AtREM1.3 taken by VA-TIRFM were shown in Fig. 1, additional movie files showed these in more detail (Additional files 1 and 2). In contrast to AtREM1.3, which was patchily localized in the plane of the PM, AtRGS1-YFP showed a more even distribution on the PM during resting state (Fig. 1A, C). Moreover, most of the mCherry-AtREM1.3 spots were mobile in the diffraction-limited mode, whereas the AtRGS1-YFP particles exhibited various motion patterns on the PM (Additional files 1 and 2). Upon JA treatment, the AtRGS1-YFP spots tended to accumulate large and bright clusters on the PM, while the fluorescence intensity of the mCherry-AtREM1.3 particles was comparable to that without JA treatment (Fig. 1B, D). We then used spatial clustering index (SCI), which represents protein lateral organization, to quantitatively analyze the cluster degree through dividing the mean of the 5% highest values by the mean of 5% lowest fluorescence intensities values in single-molecular images. In steady state, the SCI of AtRGS1-YFP was 2.16, which significantly increased to 4.74 (Additional file 3: Fig. S1). However, the SCI of mCherry-AtREM1.3 had limited change with or without JA treatment (3.42 vs. 3.56) (Additional file 3: Fig. S1).

### SM-kymograph-based quantification of PM protein dwell time

The dwell time, a key parameter of endocytosis, was defined as the time during which PM proteins clustered into the coated pits with the concerted action of multiple endocytic proteins before endocytosis, which is important for controlling signaling transmission [2, 8]. 3D images were generated by SM-based kymography to identify appropriate individual particles and improve the accuracy of the quantitative dwell-time analysis. Recent studies have demonstrated that stimulation by JA induces the endocytosis of AtRGS1 [21]. To analyze the dwell time of AtRGS1-YFP upon JA treatment, we used the imaging software ImageJ to open the series of TIFF images taken by VA-TIRFM and saved these successive frames into 16-bit format by clicking ‘Image/Stacks/Image to stacks’. The kymograph plug-in of ImageJ was used to calculate the dwell time according to the protocol described in “Methods” section.

**Fig. 2:**
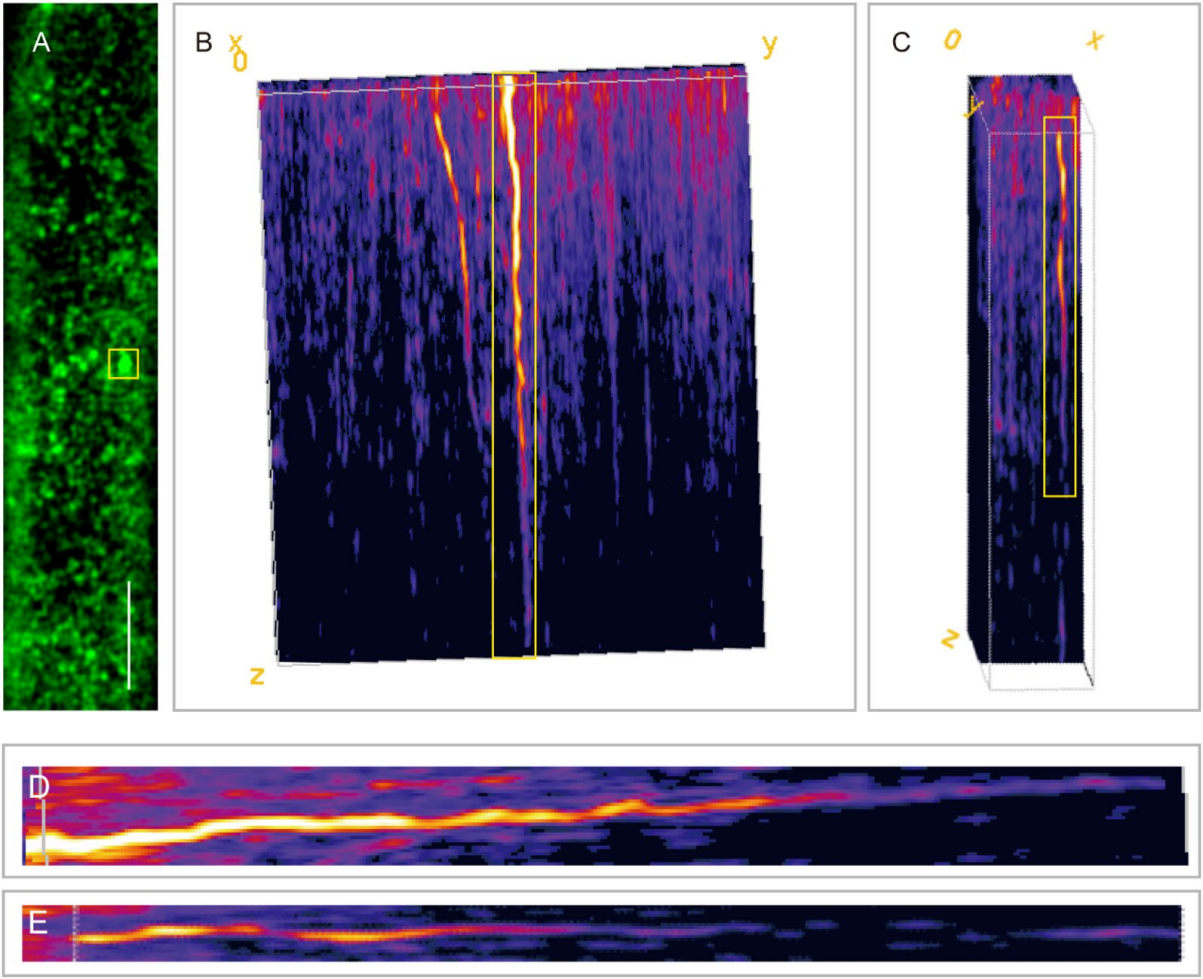
Comparison of the dwell time for the same point by SM kymography. **A** The typical single-particle images of AtRGS1-YFP in *Arabidopsis* hypocotyl cells by VA-TIRFM. Bar = 2 μm. **B**, **C** SM kymography analysis of an AtRGS1-YFP particle (the boxed area in **A**) with different tilt angles. **D**, **E** Magnified images of the yellow rectangle marked area in **B** and **C**

Unlike the traditional kymograph analysis (Additional file 4: Fig. S2), we used ImageJ to transform the 2D trajectory and timeline (2D+t) into a three-dimensional (3D) formation by ‘Plugins/3D/Volume viewer’. The beginning of the horizontal line represents the time at which the protein is on the PM, the end of the horizontal line represents the time at which the protein might be internalized, and the length of the horizontal line refers to the duration of time spent by the protein at the cell surface. First, we selected one AtRGS1-YFP fluorescent particle marked by the yellow frame from sequences without JA treatment (Fig. 2A). Notably, we found that analysis from different angles of rotation might result in different dwell time data for the same particle (Fig. 2). For example, the motion trajectory of the AtRGS1-YFP particle (x = 6; y = − 83; z = − 100) was an overlength line that exceeded the time for sequence acquired by VA-TIRFM (Fig. 2B, D), while the same point showed appearance into, and disappearance from, the focal plane when the angle (x = − 83; y = − 3; z = − 27) was adjusted by the ‘Image/Plugins/Volume Viewer’ function of ImageJ (Fig. 2C, E). Therefore, this particle was thought to be an inappropriate point for dwell time analysis and should be abandoned in the data analysis, implying that 2D kymograph analysis is not suitable for accurately calculating the dwell time. Taken together, we recommend that all interested individual points from the 3D images should be manually verified to avoid misinterpreting dynamic events before dwell time analysis.

**Fig. 3:**
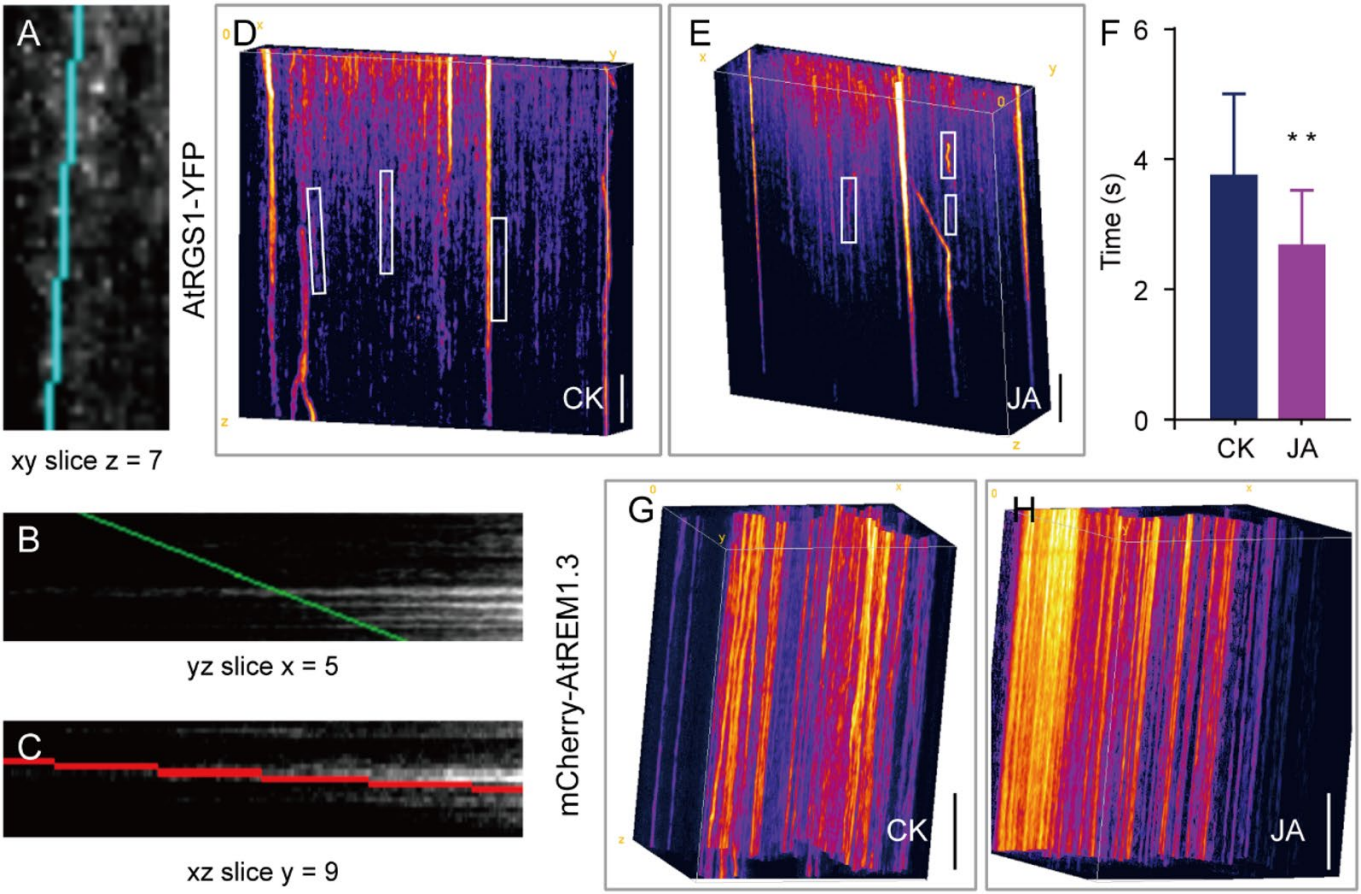
SM kymography analysis for the dwell time of AtRGS1-YFP and mCherry-AtREM1.3 under different conditions. **A**–**C** The tilt angle of **D** in xy (**A**), yz (**B**), or xz (**C**) slice. **D**, **E** The typical 3D viewer of AtRGS1-YFP with (**E**) or without JA stimulation (**D**). **F** The average dwell time of AtRGS1-YFP with or without JA treatment. **P < 0.01. Student’s *t*-test. Error bars represent the SD. n = 30. **G**, **H** The typical 3D viewer of mCherry-AtREM1.3 with (**H**) or without JA stimulation (**G**). Bar = 2 s in **D**, **E** and **G**, **H**. The 6-day-old transgenic seedlings expressing AtRGS1-YFP or mCherry-AtREM1.3 were treated with ½ MS liquid medium (CK) and 100 μM MeJA (JA) for 8 h

In accordance with the above-mentioned principle, we chose appropriate points whose trajectory lines were unchanged regardless of whether the angles of the 3D information images were adjusted or not. The representative 3D images of AtRGS1-YFP with or without JA treatment are shown in Fig. 3D–E, in which the typical motion trajectories of the AtRGS1-YFP particles are labeled with white frames. Figure 3A–C show the rotation parameters for Fig. 3D. Under a steady state, the residence times of AtRGS1-YFP on the PM averaged 3.8 s (Fig. 3F). After JA treatment, the trace of the AtRGS1-YFP particles presented a more skewed and shorter timeline (Fig. 3E), showing a significant decrease in the dwell time to 2.7 s (Fig. 3F), indicating that JA application increases the motion of the AtRGS1 particles and may induce the endocytosis of AtRGS1. In contrast to the relatively low stability of AtRGS1-YFP on the PM, the trajectories of the mCherry-AtREM1.3 particles in the 3D images were much longer with or without JA treatment (Fig. 3G, H). The dwell time of AtREM1.3 could not be measured because of the overlength line out of the images acquired by VA-TIRFM (Fig. 3G, H), suggesting that AtREM1.3 protein stays on the PM with limited trafficking.

### SM-kymograph-based analysis of the colocalization of different proteins

PM proteins always form stable or transient complexes to regulate various biological processes. The degree of colocalization between two different PM proteins is an important parameter for assessing complex formation [16–21]. Traditional technologies frequently overestimate the colocalization values because of the indeterminate parameters of the ‘length width’ plug-in. The SM kymograph analysis provides high spatiotemporal information for colocalization with high dimensionality.

**Fig. 4:**
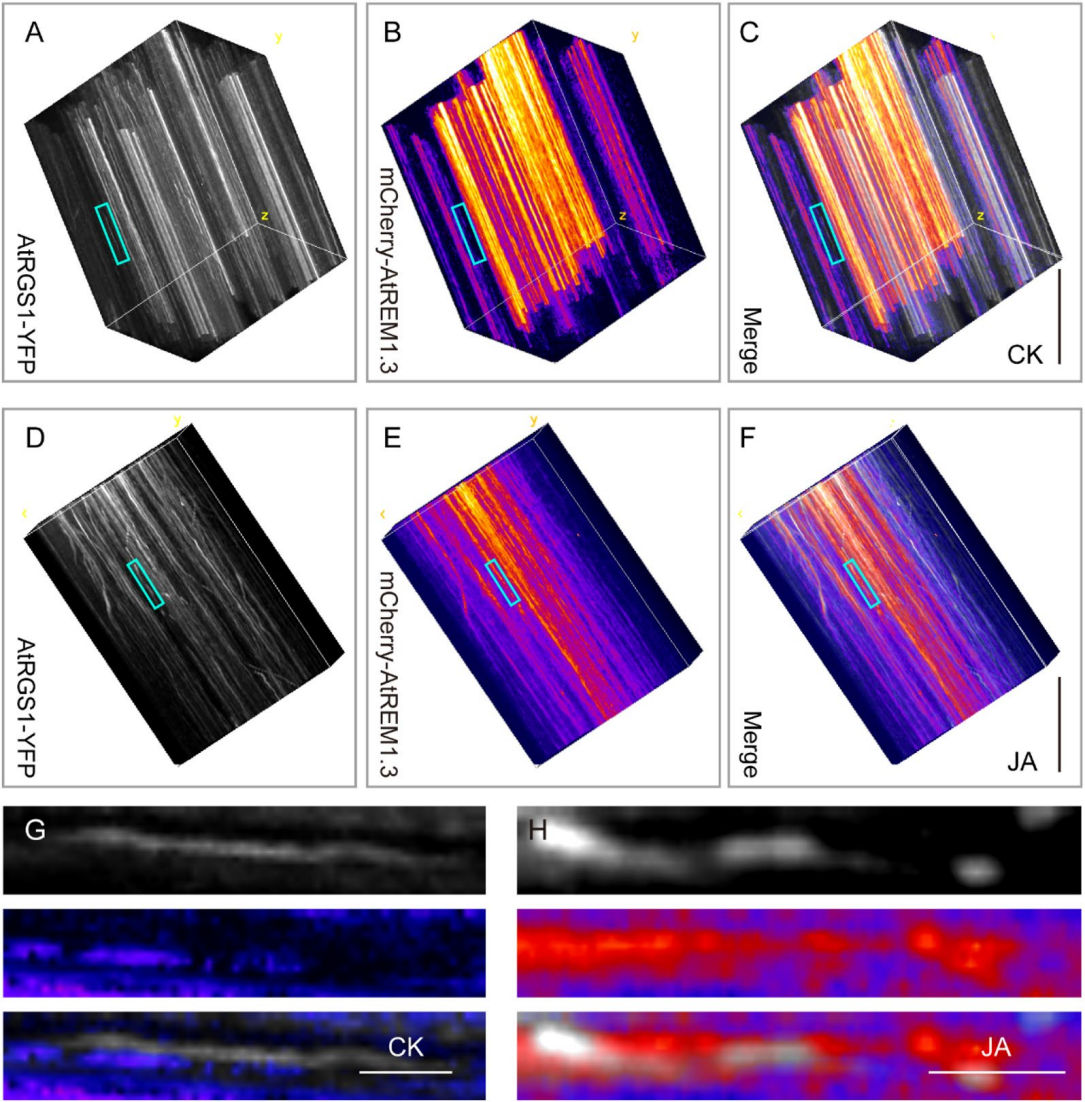
SM kymography analysis for the colocalization between AtRGS1-YFP and mCherry-AtREM1.3 under different conditions. **A**–**C** The typical 3D viewer of AtRGS1-YFP (**A**), mCherry-AtREM1.3 (**B**), and merge (**C**) images at steady state. Bar = 5 s. **D**–**F** The typical 3D viewer of AtRGS1-YFP (**D**), mCherry-AtREM1.3 (**E**), and merge (**F**) images with JA treatment. Bar = 5 s. **G** The magnified images of the blue rectangle marked areas in **A**–**C**. Bar = 1 s.** H** The magnified images of the blue rectangle marked areas in **D**–**F**. Bar = 1 s. The 6-day-old transgenic seedlings co-expressing AtRGS1-YFP and mCherry-AtREM1.3 were treated with ½ MS liquid medium (CK) and 100 μM MeJA (JA) for 8 h

A previous study revealed that the agonist-targeted endocytosis of AtRGS1 is dependent on lipid rafts [22]. To examine whether JA also regulates motion changes of AtRGS1 particles through lipid rafts, we used SM kymography to investigate the colocalization of AtRGS1-YFP and mCherry-AtREM1.3 by coexpression analysis in transgenic *Arabidopsis* seedlings (Fig. 4). Notably, the expression levels of these two fluorescent-labeled proteins should be as consistent as possible. After obtaining the bifluorescence images by VA-TIRFM, we used MatLab software to correct the model error between the GFP and mCherry images by ‘MatLab/Croplmage4color.m/Process files’ and saved them as new images. The traditional kymograph analysis for tracking the colocalization degree of the PM protein–protein interactions in 2D images is shown in Additional file 5: Fig. S3 and Additional file 6: Fig. S4. In the SM kymograph method, these 2D+t images were transformed into 3D formation through ‘Plugins/3D/Volume viewer’ in ImageJ. Next, we adjusted the angle of the 3D images of AtRGS1-YFP and mCherry-AtREM1.3 according to the principle of the dwell time analysis mentioned above and merged these two images using ‘Image/Color/Merge channels’ in ImageJ.

Under control condition, the trajectories of AtRGS1-YFP and mCherry-AtREM1.3 always exhibited two independent traces (Fig. 4A–C, G), indicating that the AtREM1.3-labelled lipid raft has limited function in changing the motion of AtRGS1. Upon JA treatment, the overlap traces between AtRGS1-YFP and mCherry-AtREM1.3 obviously increased, implying that the AtREM1.3-labelled lipid raft may contribute to JA-induced AtRGS1 internalization (Fig. 4D–F, H). For example, at t = 2 s, the AtRGS1-YFP fluorescence appeared and colocalized with the pre-existed mCherry-AtREM1.3 signal (Fig. 4H). Then, the AtRGS1-YFP trace continued until it disappeared 2 s later, while the mCherry-AtREM1.3 trace still lasted (Fig. 4H). These results suggest that JA induces the accumulation of AtRGS1 into an AtREM1.3-dependent pool on the PM before its endocytosis.

## Discussion

Kymography is a useful strategy for analyzing the motion behaviors of objects of interest, as its records the dynamics of the spatial dimensions [2, 7–13]. Several types of kymography have been applied in animal and yeast cells to reveal the dynamic behaviors of hyperfine structures, such as KymographClear, KymographDirect, KymoButler and Kymolyzer [23–25]. However, the high density of plant PM proteins often results in excessive blurry spots when analyzed using traditional fluorescence microscopy. Dissecting the time-normalized intensity profiles of plant PM proteins remains a challenge.

In this study, we combined VA-TIRFM and kymography to develop an SM kymograph method for the analysis of PM protein dynamics in living plant cells. Successive images acquired by VA-TIRFM at the microsecond and nanometer scales were used to accurately analyze the dwell time and colocalization of plant PM proteins by kymography. We also recommend photo-switchable fluorescent probes instead of traditional fluorescein for high-density interest proteins in plants. Monomeric Eos fluorescent protein appears to be an attractive candidate that is activated by violet-blue light in sparse numbers over time to overcome the current limitations of PM protein motion features.

The dwell time of PM proteins is tightly associated with signal transmission in response to environmental stimuli [8]. Based on 2D information, the dwell time analyzed by traditional kymography might be miscalculated. SM kymograph analysis offers a line-scan technique that can be used to accurately examine the dwell time of PM proteins in living cells from the volume-velocity output of 3D images, which can advance the understanding of PM protein flow features in response to extra- or intracellular-signaling. The degree of colocalization, which reflects the relationship between different proteins, is widely used to illustrate the possibility of protein–protein interactions [2, 20]. Colocalization analysis is typically based on conventional fluorescence microscopies with 2D images, which is frequently inaccurate because of the lack of dynamic information in an unabridged timeline direction. The SM kymograph analysis generates 3D images that contain 2D+t information to trace the trajectories from the specific fluorescein at millisecond intervals and provides accurate measurements of the colocalization ratio by adjusting the rotation angle at the spatio-temporal level.

## Conclusions

In this study, SM kymograph analysis provided a powerful method for analyzing plant PM protein dynamics, such as endocytosis and interaction, based on dwell time and protein–protein colocalization degree, respectively. In SM kymograph, VA-TIRFM is commonly used in the observation of PM proteins and ImageJ is a classical program dedicated to analyzing image data. Here, we selected two representative PM proteins with distinct membrane anchoring mode and dynamic behaviors, AtRGS1 and AtREM1.3, to verify the feasibility of SM kymograph analysis. Thus, SM kymograph could be applied to different PM proteins and might be used in a wide range of systems to record the mobility of highly fine structures in any cell types.

## Methods

### Plant materials and growing conditions

To observe the dynamics of proteins in living plant cells, the interests should be labeled with fluorophores tags that are ideally resistant to photobleaching with a significant quantum yield. EGFP is one of the widely utilized fluorescent tags in VA-TIRF microscopy analysis, which transforms traditional GFP with K replaced A in 206 [26]. It is important to note that the biological function of fluorescent-labeled proteins should be confirmed by genetic complementary experiments, and these proteins’ expression level should be detected by Western Blotting. The transgenic plants which have functional. Fluorescent-labeled proteins with similar expression levels to wild-type plants could be used for further analysis.

½ Murashige and Skoog medium (2.21 g of MS salts (PhytoTechnology Laboratories, cat. no. M519) per liter; pH 5.6–5.8) with no sugar was sterilized for 15 min at 121 °C before using. *Arabidopsis* seeds were surface-sterilized successively with 90% ethanol for 5 min and 70% ethanol for 10 min, then washed 4 times using sterile water. After being placed in 12-well cell culture plates (Corning, cat. no. 3513) with liquid ½ MS medium, seeds were chilled at 4 °C for 2 days in darkness. After that, seeds were placed on a horizontal shaker in a growth room at 22 °C with light for 2 h to induce germination firstly and keep in dark for 6 days before imaging. For JA treatment, 6-day-old seedlings were transferred to 100 μM JA-supplemented ½ MS liquid medium and imaged after 8 h.

### VA-TIRFM and single-particle fluorescence image analysis

The laser intensity of VA-TIRFM is a central parameter for minimizing errors caused by the photobleaching of fluorescence dye [26]. We recommend that 15% of the laser intensity applied in transgenic seedlings is enough to activate fluorophores and impede fast photobleaching. The EM gain of the CCD camera is manipulated for producing an optimum signal-to-noise ratio to detect moving proteins above background signals [27]. We recommend 10–50 Hz and 100–350 ms to track single granule signals, 200 frames for data quantification. Data analysis can be performed on standard 32-bit or 64-bit hardware and exceeding 4 GB of memory is required according to image frames and particle numbers, noting that the TIFF format of microscopy data is applicable for analysis in ImageJ. For the analysis of protein dynamic, the first 100 frames of one serial image were used to track the trajectories of the interest protein using ImageJ software. The machine learning workflow and software download instructions are available on U-track (http://lccb.hms.harvard.edu/software.html) and Fluoro-Bancroft (http://physics-server.uoregon.edu/~raghu/particle_tracking.html).

Special coverslip (sizes: 24 mm × 50 mm; thickness: 0.13 mm; Menzel-Gläser: cat. no. BB024024A1) with better transparency and refractive index is suitable to reach total reflection of light transmittance [26, 27]. Under the premise of avoiding seedling damage and ensuring cell viability, two pieces of bearing blocks were placed on both edges of the small coverslip to increase load, as a simple and efficient method for improving the refractive index.

### Kymograph analysis

The origin images were uploaded to ImageJ which contains the ‘multiple kymograph’ plugin (Fiji, 2.0.0-rc-65/1.51w). The contrast of images is adjusted by clicking ‘Image/Adjust /Brightness/Contrast’, then clicking ‘Auto’ for optimal contrast. Subsequently, region of interests (ROI) in download images are manually outlined using an arbitrarily sized rectangle selection tool and saved in an appointed file by clicking ‘Image/Crop’ (for example, if the transgenic seedlings have different fluorescent labels or different treatments, save them respectively). The dwell-time and motion state of ROI particles are quantitated by the ‘Analyze/Set scale’ tool. The ‘Known distance’ is set as a special value according to the magnification times of raw images and the resolution of VA-TIRFM. After the interest single particles are generated in the ‘straight line’ tool, ‘Plugins/Multiple Kymograph’ is used to analyze the dynamic features. With a following emerged pop-up, choose the appropriate value according to particle characteristics in ‘Linewidth’ (must be odd numbers, while ‘11’ is the best choice for most particles in plant cells). Finally, the ‘Image/Type/RGB color’ tool is used to gain typical images followed by TIFF format saving for accurately calculating dwell-time and relevance of different proteins by the FIJI measure tool. At least three seedlings were taken for imaging in each treatment, and three different positions were chosen in individual plants.

## Supplementary Information

**Additional file 1.** VA-TIRFM imaging of AtRGS1-YFP particles at the PM of Arabidopsis hypocotyl cells.

**Additional file 2.** VA-TIRFM imaging of mCherry-AtREM1.3 particles at the PM of Arabidopsis leaves epidermal cells.

**Additional file 3: Figure S1.** SCI of AtRGS1-YFP or mCherry-AtREM1.3 analyzed by ImageJ software under different conditions. The 6-day-old transgenic seedlings expressing AtRGS1-YFP (**A**) and mCherry-AtREM1.3 (**B**) were treated with ½ MS liquid medium (CK) and 100 μM MeJA (JA) for 8 h by TIRFM observation. ***P < 0.001. ns > 0.05. Student’s *t*-test. Error bars represent the SD. n = 32 to 67 per treatment.

**Additional file 4: Figure S2.** Dwell time distribution of AtRGS1-YFP and mCherry-AtREM1.3 on the PM analyzed by traditional kymography methods under different conditions. The 6-day-old transgenic seedlings expressing AtRGS1-YFP (**A**, **B**) and mCherry-AtREM1.3 (**C**, **D**) were treated with ½ MS liquid medium (CK) and 100 μM MeJA (JA) for 8 h. Bar = 2 s.

**Additional file 5: Figure S3.** Colocalization between AtRGS1-YFP and mCherry-AtREM1.3 analyzed by VA-TIRFM under different conditions. **A**–**C** Typical single-molecular viewer of AtRGS1-YFP (**A**), mCherry-AtREM1.3 (**B**), and merge (**C**) images at steady state. **D**–**F** Typical single-molecular viewer of AtRGS1-YFP (**D**), mCherry-AtREM1.3 (**E**), and merge (**F**) images upon JA treatment. Bar = 5 μm. The 6-day-old transgenic seedlings co-expressing AtRGS1-YFP and mCherry-AtREM1.3 were treated with ½ MS liquid medium (CK) and 100 μM MeJA (JA) for 8 h.

**Additional file 6: Figure S4.** Colocalization between AtRGS1-YFP and mCherry-AtREM1.3 analyzed by traditional kymography under different conditions. **A**–**C** Typical 2D viewer of AtRGS1-YFP (**A**), mCherry-AtREM1.3 (**B**), and merge (**C**) images at steady state. **D**–**F** Typical 2D viewer of AtRGS1-YFP (**D**), mCherry-AtREM1.3 (**E**), and merge (**F**) images with JA treatment. Bar = 3 s. The 6-day-old transgenic seedlings co-expressing AtRGS1-YFP and mCherry-AtREM1.3 were treated with ½ MS liquid medium (CK) and 100 μM MeJA (JA) for 8 h.

## Data Availability

All data and materials are available upon request to XS (shanxy80@tsinghua.edu.cn).

## Abbreviations

SM: Single molecular
PM: Plasma membrane
WT: Wild-type
VA-TIRFM: Variable angle-total internal reflection fluorescence microscopy
SPT: Single-particle (co-)tracking
