# Supplementary material for: VA-TIRFM-based SM kymograph analysis for dwell time and colocalization of plasma membrane protein in plant cells
Source: Plant Methods. 2023 Jul 8;19:70. doi: 10.1186/s13007-023-01047-5 (PMC10329380; doi:10.1186/s13007-023-01047-5)
Supplement: Supplementary file 3 — Additional file 3: Figure S1. SCI of AtRGS1-YFP or mCherry-AtREM1.3 analyzed by ImageJ software under different conditions. The 6-day-old transgenic seedlings expressing AtRGS1-YFP (A) and mCherry-AtREM1.3 (B) were treated with ½ MS liquid medium (CK) and 100 μM MeJA (JA) for 8 h by TIRFM observation. ***P < 0.001. ns > 0.05. Student’s t-test. Error bars represent the SD. n = 32 to 67 per treatment. [file 13007_2023_1047_MOESM3_ESM.pdf]

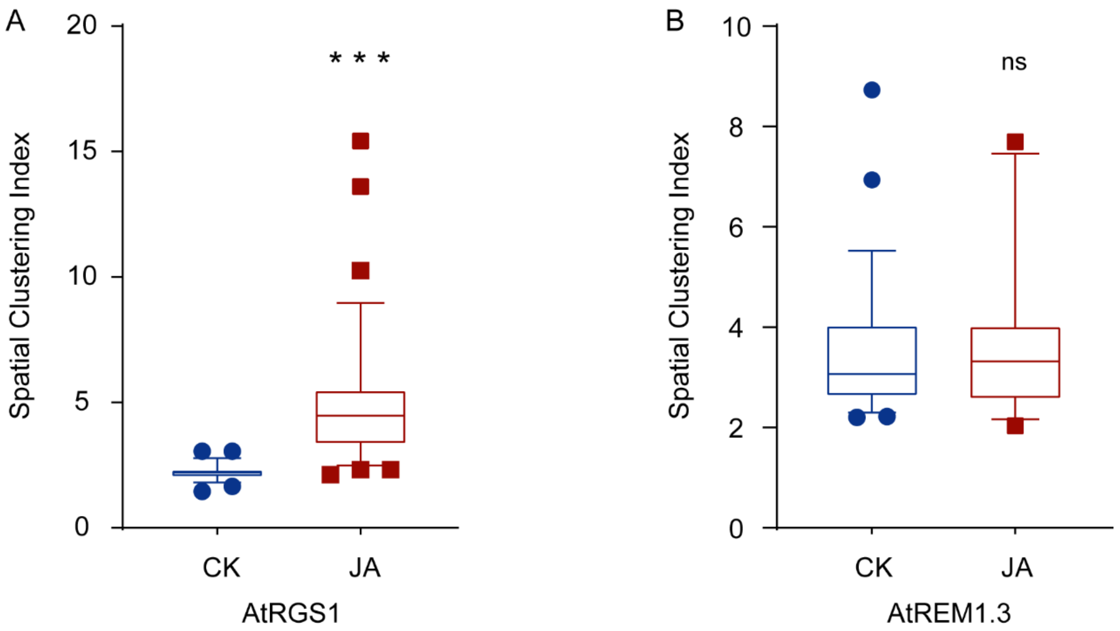

Additional file 3: Fig. S1 SCI of AtRGS1-YFP or mCherry-AtREM1.3 analyzed by ImageJ software under different conditions. The 6-day-old transgenic seedlings expressing AtRGS1-YFP (**A**) and mCherry-AtREM1.3 (**B**) were treated with ½ MS liquid medium (CK) and 100 μM MeJA (JA) for 8 h by TIRFM observation. ***$P < 0.001$. ns > 0.05. Student's *t*-test. Error bars represent the SD. n = 32 to 67 per treatment.
