# Supplementary material for: VA-TIRFM-based SM kymograph analysis for dwell time and colocalization of plasma membrane protein in plant cells
Source: Plant Methods. 2023 Jul 8;19:70. doi: 10.1186/s13007-023-01047-5 (PMC10329380; doi:10.1186/s13007-023-01047-5)
Supplement: Supplementary file 4 — Additional file 4: Figure S2. Dwell time distribution of AtRGS1-YFP and mCherry-AtREM1.3 on the PM analyzed by traditional kymography methods under different conditions. The 6-day-old transgenic seedlings expressing AtRGS1-YFP (A, B) and mCherry-AtREM1.3 (C, D) were treated with ½ MS liquid medium (CK) and 100 μM MeJA (JA) for 8 h. Bar = 2 s. [file 13007_2023_1047_MOESM4_ESM.pdf]

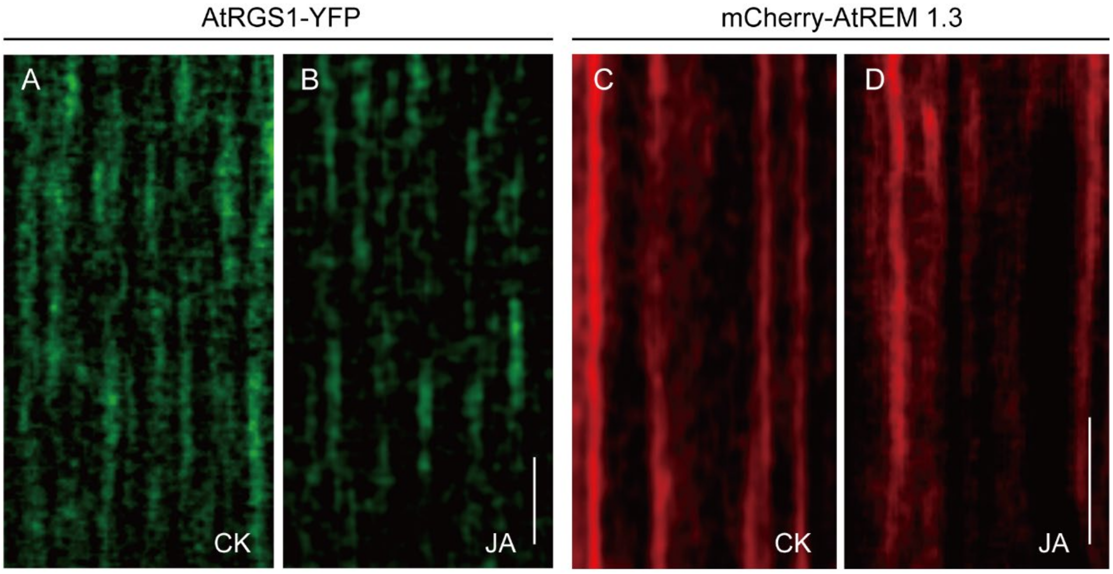

Additional file 4: Figure S2. Dwell time distribution of AtRGS1-YFP and mCherry-AtREM1.3 on the PM analyzed by traditional kymography methods under different conditions. The 6-day-old transgenic seedlings expressing AtRGS1-YFP (**A, B**) and mCherry-AtREM1.3 (**C, D**) were treated with ½ MS liquid medium (CK) and 100 μM MeJA (JA) for 8 h. Bar = 2 s.
