# Supplementary material for: VA-TIRFM-based SM kymograph analysis for dwell time and colocalization of plasma membrane protein in plant cells
Source: Plant Methods. 2023 Jul 8;19:70. doi: 10.1186/s13007-023-01047-5 (PMC10329380; doi:10.1186/s13007-023-01047-5)
Supplement: Supplementary file 6 — Additional file 6: Figure S4. Colocalization between AtRGS1-YFP and mCherry-AtREM1.3 analyzed by traditional kymography under different conditions. A–C Typical 2D viewer of AtRGS1-YFP (A), mCherry-AtREM1.3 (B), and merge (C) images at steady state. D–F Typical 2D viewer of AtRGS1-YFP (D), mCherry-AtREM1.3 (E), and merge (F) images with JA treatment. Bar = 3 s. The 6-day-old transgenic seedlings co-expressing AtRGS1-YFP and mCherry-AtREM1.3 were treated with ½ MS liquid medium (CK) and 100 μM MeJA (JA) for 8 h. [file 13007_2023_1047_MOESM6_ESM.pdf]

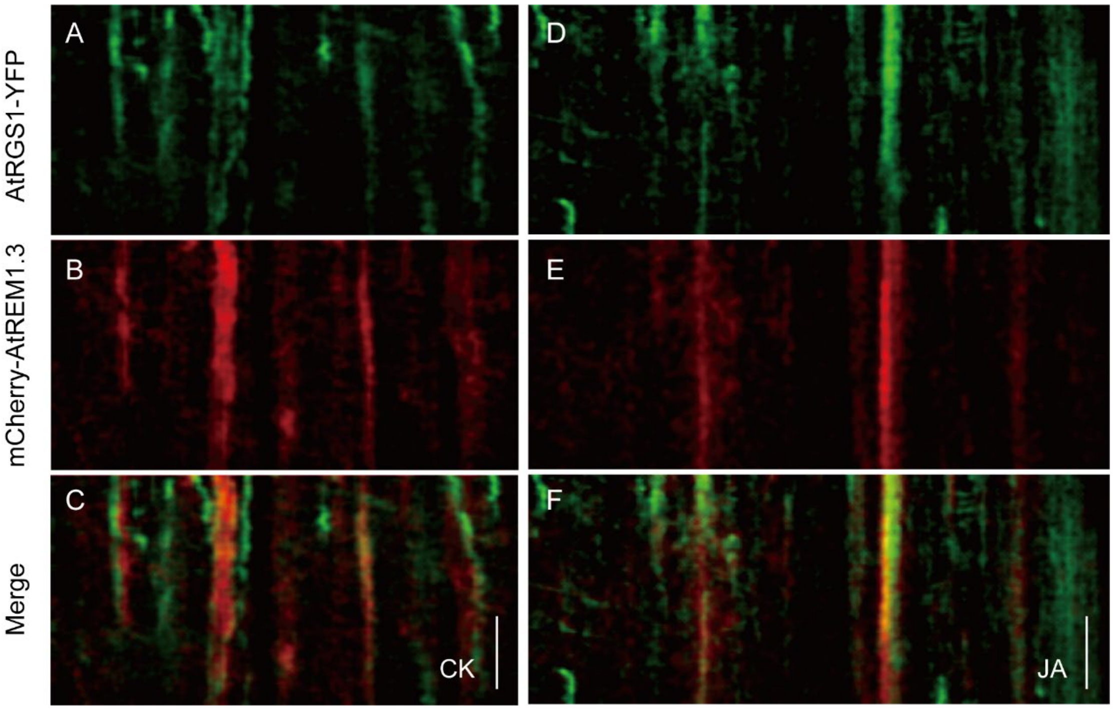

Additional file 6: Fig. S4 Colocalization between AtRGS1-YFP and mCherry-AtREM1.3 analyzed by traditional kymography under different conditions. **A–C** Typical 2D viewer of AtRGS1-YFP (**A**), mCherry-AtREM1.3 (**B**), and merge (**C**) images at steady state. **D–F** Typical 2D viewer of AtRGS1-YFP (**D**), mCherry-AtREM1.3 (**E**), and merge (**F**) images with JA treatment. Bar = 3 s. The 6-day-old transgenic seedlings co-expressing AtRGS1-YFP and mCherry-AtREM1.3 were treated with ½ MS liquid medium (CK) and 100 μM MeJA (JA) for 8 h.
